# Mu-Opioid Receptors Transiently Activate the Akt-nNOS Pathway to Produce Sustained Potentiation of PKC-Mediated NMDAR-CaMKII Signaling

**DOI:** 10.1371/journal.pone.0011278

**Published:** 2010-06-23

**Authors:** Pilar Sánchez-Blázquez, María Rodríguez-Muñoz, Javier Garzón

**Affiliations:** 1 Neuropharmacology, Cajal Institute, CSIC, Madrid, Spain; 2 CIBER of Mental Health (CIBERSAM) G09, ISCIII, Madrid, Spain; Universidade Federal do Rio de Janeiro (UFRJ), Brazil

## Abstract

**Background:**

In periaqueductal grey (PAG) matter, cross-talk between the Mu-opioid receptor (MOR) and the glutamate N-methyl-D-Aspartate receptor (NMDAR)-CaMKII pathway supports the development of analgesic tolerance to morphine. In neurons, histidine triad nucleotide binding protein 1 (HINT1) connects the regulators of G protein signaling RGSZ1 and RGSZ2 to the C terminus of the MOR. In response to morphine, this HINT1-RGSZ complex binds PKCγ, and afterwards, the interplay between PKCγ, Src and Gz/Gi proteins leads to sustained potentiation of NMDAR-mediated glutamate responses.

**Methodology/Principal Findings:**

Following an intracerebroventricular (icv) injection of 10 nmol morphine, Akt was recruited to the synaptosomal membrane and activated by Thr308 and Ser473 phosphorylation. The Akt activation was immediately transferred to neural Nitric Oxide Synthase (nNOS) Ser1417. Afterwards, nitric oxide (NO)-released zinc ions recruited PKCγ to the MOR to promote the Src-mediated phosphorylation of the Tyr1325 NMDAR2A subunit. This action increased NMDAR calcium flux and CaMKII was activated in a calcium-calmodulin dependent manner. CaMKII then acted on nNOS Ser847 to produce a sustained reduction in NO levels. The activation of the Akt-nNOS pathway was also reduced by the binding of these proteins to the MOR-HINT1 complex where they remained inactive. Tolerance to acute morphine developed as a result of phosphorylation of MOR cytosolic residues, uncoupling from the regulated G proteins which are transferred to RGSZ2 proteins. The diminished effect of morphine was prevented by LNNA, an inhibitor of nNOS function, and naltrindole, a delta-opioid receptor antagonist that also inhibits Akt.

**Conclusions/Significance:**

Analysis of the regulatory phosphorylation of the proteins included in the study indicated that morphine produces a transient activation of the Akt/PKB-nNOS pathway. This activation occurs upstream of PKCγ and Src mediated potentiation of NMDAR activity, ultimately leading to morphine tolerance. In summary, the Akt-nNOS pathway acts as a primer for morphine-triggered events which leads to the sustained potentiation of the NMDAR-CaMKII pathway and MOR inhibition.

## Introduction

The coexistence of ligand-regulated metabotropic and ionotropic receptors in the postsynapse raises the possibility of mutual interactions triggered by the relative abundance of their presynaptic mediators. Among ligand-gated ionotropic receptors, glutamate N-methyl-D-Aspartate receptors (NMDARs) have received particular attention because of their crucial roles in excitatory synaptic transmission, plasticity, and neurodegeneration [1]. NMDARs are composed of NR1, NR2 (A, B, C, and D) and NR3 (A and B) subunits. They form a tetramer consisting of a pair of NR1 subunits associated with at least one type of NR2/3 subunits [2]. The NMDAR controls a cation channel that is highly permeable to Ca^2+^. In general, G-protein coupled receptors (GPCRs) regulate NMDAR activity through the action of non-receptor tyrosine kinases including Src, Fyn and serine/threonine kinases such as protein kinase C (PKC) [3]. Non-receptor tyrosine kinases phosphorylate specific tyrosine residues in the cytosolic tail of NR2 and, potentially, NR3 subunits [4], producing an increase in the permeation of Ca^2+^ ions towards the cytosolic side of the postsynapse. PKC is essential to the potentiation of NMDARs. This kinase enhances the activity of Src [5]; PKC promotes the regulatory phosphorylation of serine and threonine residues in the cytosolic regions of NR1 and NR2 subunits [6]–[8]; it directly elicits the potentiation of NMDAR responses by removing the inhibitory binding of Ca-Calmodulin (Ca^2+^-CaM) on NR1 subunits [9].

The Mu-opioid receptor (MOR) is a well-characterized GPCR in terms of its regulation by NMDARs. MOR signaling can be modulated by the NMDAR/NO cascade [10], [11], and the development of morphine-induced desensitization is a consequence of the activity of glutamate NMDARs [12], [13]. The interaction between these receptors is bidirectional, and MOR signaling in the brainstem and medulla causes a sustained increase in glutamate-activated NMDAR currents [14], [15]. Thus, the attenuation of opioid efficacy that is observed in certain states of persistent pain is caused by an anomalous increase in NMDAR function [16]. This cross-regulation also provides the molecular basis for understanding the clinical efficacy of NMDAR antagonists in opioid-induced tolerance [17], [18]. The supraspinal analgesic effects of intracerebroventricular (icv)-injected opioids are mediated through their binding to MORs located in the periaqueductal grey matter (PAG) of the midbrain [19], [20]. The rostral ventromedial medulla (RVM) which includes the nucleus raphe magnus and the laterally adjacent reticular formation is the major relay of midbrain PAG neurons that project down to the substantia gelatinosa in the dorsal horn of the spinal cord. There, these projections reduce the intensity of the ascending nociceptive signals [21], [22]. This midbrain system also participates in the antinociceptive effects of systemic morphine. Thus, lesions in the medulla or spinal cord that affect descending projections from the RVM as well as opioid antagonists delivered into the RVM diminish the effects of systemic morphine [21]. In humans, icv morphinotherapy is a very effective treatment for chronic intractable pain of cancerous origin [23]. Also, deep brain stimulation targeting PAG has been used to treat intractable pain for over 50 years. The analgesia produced by electrical stimulation of PAG is mediated by the release of endogenous opioids acting at MORs [24], and it has been successful in alleviating phantom limb pain, post-herpetic neuralgia, anesthesia dolorosa, brachial plexus injury and diverse neuropathies [25], [26].

Therefore, the PAG is of particular physiological relevance in the nociceptive modulating network that operates both at a supraspinal level and also through dorsal horn interneurons. In recent studies, it has been shown that the MOR carries a signaling complex in its C terminus that is integrated by HINT1 and RGSZ proteins [27], [28]. In response to morphine, NMDAR-nNOS generated zinc recruits PKCγ to the HINT1-RGSZ complex. Together with Src and MOR-activated Gi and Gz proteins, the complex integrates the molecular machinery that carries signals from MOR to the NMDAR [11], [29]. Cross-regulation between MORs and NMDARs creates a negative feedback loop. As a result, NMDAR inhibits MOR signaling [30]. Although pharmacological approaches have identified a series of signaling proteins involved in this process, little is known concerning whether they operate in the same regulatory machinery, and also the chronological order in which they are activated. There is a possibility, however, that MOR may regulate other events upstream of PKC and Src activation as well.

The phosphoinositide 3-kinase (PI3K) cascade has been associated with MOR desensitization [31]. Akt, a 57 kDa serine/threonine protein kinase, also known as protein kinase B or Rac, mediates the effects of PI3K [32]. MOR activates Akt via Gi/o and PI3K [33], [34] as well as regulating Akt expression in neural cells [35], [36]. Akt contains a pleckstrin homology (PH) domain at its N-terminus followed by a kinase domain and a carboxy terminal regulatory domain. The PH domain, which contains approximately 120 amino acids, can be found in a wide range of proteins involved in intracellular signaling. This domain can bind phosphatidylinositol lipids within biological membranes, such as phosphatidylinositol (3,4,5)-triphosphate (PIP3) and phosphatidylinositol (4,5) biphosphate (PIP2). The nNOS pathway has also been linked to the development of morphine tolerance [37]. Interestingly, NMDAR signaling elicits nNOS activation via the PI3K-Akt pathway [38].

The PAG is of relevance in the treatment of chronic pain in humans and it is essential in the analgesic effects produced by icv and systemic opioids. Thus, we wanted to analyze in this neural structure the connection between morphine-activated MORs and the Akt-nNOS pathway. Because the HINT1 protein is required for morphine to recruit PKCγ to the MOR environment, we evaluated in HINT1 (−/−) mice this possibility but with Akt and nNOS. We also evaluated the inhibition of Akt and nNOS enzyme activities on the morphine-induced transient associations observed between MORs and signaling proteins as well as in the development of analgesic tolerance to morphine.

## Materials and Methods

### Animals, intracerebroventricular injection and evaluation of antinociception

Male albino CD-1 mice weighing 22–25 g were used throughout the study. The mice were housed and used in accordance with the guidelines of the European Community for the Care and Use of Laboratory Animals (Council Directive 86/609/EEC). This research has been approved by the Bioethics Committee of the “Consejo Superior de Investigaciones Científicas (CSIC)”. The animals were housed at 22°C under a 12 h light/dark cycle (lights on from 8 a.m. to 8 p.m.). Food and water were provided ad libitum. Response to nociceptive stimuli was determined with a warm water (52°C) tail-flick test. Baseline latencies ranged from 1.5 to 2.2 seconds. These latencies were not significantly affected by naltrindole (Tocris 0740) or the nNOS inhibitor L-NG-Nitroarginine (LNNA, Tocris 0664). A cut-off time of ten seconds was implemented to minimize the risk of tissue damage. Antinociception was expressed as a percentage of the maximum possible effect (MPE), where MPE  = 100 x (test latency (s) - baseline latency (s))/(cut-off time 10 s-baseline latency (s)). Groups of eight to ten mice were lightly anesthetized with ether and injected with a 10 nmol dose (4 µL total volume) of morphine sulfate (Merck, Darmstadt, Germany) into the lateral ventricle. Following the injection, antinociception was assessed at several time points.

The tail-flick analgesic test applies a thermal noxious stimulus to promote flicking of the mouse's tail, and opioids increase the time elapsed between application of the stimulus and the flick. This response comprises a spinal reflex which is under facilitator drive by the brain stem nociceptive modulating network [39]. After icv administration of opioids, the MORs in the ventral region of PAG [40] play an important role in the supraspinal pathways that modulate spinal nociceptive processing [41]. Thus, icv morphine modulates descending serotoninergic and adrenergic systems and inhibits responses to nociceptive stimuli, including nociceptive withdrawal reflexes that are organized segmentally, such as the hind limb withdrawal and tail flick reflexes [42]. The icv doses of the opioid required to produce these effects affect regions with poor density of MORs such as the ventricle walls, the PAG which is responsible of MOR-mediated analgesia, and to a minor extent the floor of the 4^th^ ventricle without reaching the spinal level [43].

Our molecular studies that analyzed the interaction of signaling proteins with the MOR were performed in HINT1 (+/+) and HINT1 (−/−) mice (128SvJ strain) [44]. Breeding pairs of homozygous wild type (WT) and knockout (KO) mice, generously supplied by Drs. I.B. Weinstein and J.B. Wang, were obtained from hybrid mutant mice, originally created on a 129SvJ-C57BL/6 background, by backcrossing mice from the 129 strain for several generations. Thus, 96% of the genetic background of the mice used in the present study comes from the 129 strain. Animals used in this study were 8 to12 weeks-old adult male mice. The genotype was confirmed with PCR analysis of DNA extracted from tail biopsies. The absence of the HINT1 protein results in a phenotype with behavioral and endocrine features that indicate changes in the animals' mood [45].

### Production of acute tolerance and the interval required to recover the analgesic response

The animals received an icv priming dose of either 3 nmol or 10 nmol morphine in the right lateral ventricle. A 3 nmol dose produced 40–50% of the maximum analgesic effect. A 10 nmol dose, on the other hand, produced 70–80% of the maximum analgesic effect. Controls were injected only with the opioid priming dose, whereas the experimental group received either naltrindole or LNNA prior to the morphine priming dose. Mice from the control and experimental groups were divided into sub-groups of eight mice each and icv-injected with increasing test doses of morphine 24 h after the priming dose. The effect of morphine was evaluated at 30 min post-injection; allowing time for the compound to reach its peak analgesic effect. Development of acute tolerance was ascertained by constructing an analgesic dose-response curve 24 h post-injection. At this point, the analgesic effect of the 10 nmol priming dose had dissipated as evidenced by the restoration of baseline latencies in the tail-flick test. Data are expressed as the mean ± SEM from groups of eight mice.

### Co-immunoprecipitation of signaling proteins

To compare the analgesic effects with molecular events, we examined the PAG, a discrete neural structure. Synaptosomal membranes were obtained from groups of six to ten mice that were sacrificed by decapitation at various intervals following an icv injection of 10 nmol morphine. PAG tissue was collected and homogenized in 10 volumes of 25 mM Tris-HCl (pH = 7.4) and 0.32 M sucrose. The homogenized mixture was supplemented with a phosphatase inhibitor mixture (Sigma, P2850), protein kinase inhibitor H89 (Sigma, B1427) and a protease inhibitor cocktail (Sigma, P8340). The homogenate was centrifuged at 1000g for 10 min to remove the nuclear fraction. Then, the synaptosomal fraction was isolated [46], [47]. The final pellet was diluted in Tris buffer and supplemented with a mixture of protease inhibitors (0.2 mM phenylmethylsulphonyl fluoride, 2 µg/mL leupeptin, and 0.5 µg/mL aprotinin) before it was aliquoted and frozen at −80°C.

The membranes (0.5 g) were sonicated (two cycles of 5 s each) in a 1 mL mixture containing 50 mM Tris-HCl (pH = 7.7), 50 mM NaCl, 1% Nonidet P-40, 50 µL of protease and phosphatase inhibitor mixtures, as well as H89. Affinity-purified IgGs against the second extracellular loop of the MOR and the C-terminus of RGSZ2 proteins were labeled with biotin (Pierce #21217 & 21339). The target proteins were then immunoprecipitated from solubilized membranes as described previously [46], [48]. At the end of the procedure, proteins in the soluble fraction were concentrated in centrifugal filter devices (Amicon Microcon YM-10 #42407, Millipore) and solubilized by heating the samples at 100°C for 10 min in 2x Laemmli buffer containing mercaptoethanol. Once the samples cooled, the proteins were resolved by SDS-PAGE using 10–16% gels.

### Analysis of MOR phosphorylation

Because unidentified proteins might co-precipitate with the MORs and interfere with phosphoserine analysis (clone 1C8, Calbiochem, 525281), existing protein interactions were disrupted under denaturing conditions prior to performing immunoprecipitation. PAG synaptosomal membranes prepared in presence of phosphatase inhibitor mixture (Sigma, P2850), protein kinase inhibitor H89 (Sigma, B1427) and a protease inhibitor cocktail (Sigma, P8340) were heated in 40 mM Tris–HCl, 1% SDS buffer, 2-mercaptoethanol for 10 min at 100°C. The mixture was cooled to room temperature and the SDS concentration was reduced by adding octylthioglucoside to a final concentration of 0.65%. MOR was immunoprecipitated with biotinylated IgGs and after removing the IgGs the absence of accompanying proteins was verified by silver staining. The MOR detection procedure was continued as described above.

### Detection of signaling proteins in mouse brain: electrophoresis and immunoblotting

Western blots were probed with affinity-purified IgG antibodies directed against: murine MOR (1∶1000 dilution) [46]; Gαi2, Gαz (1∶2000) [49]; RGS17(Z2) [46], [50]; NMDAR1 (1∶1000, Abcam ab1880); NMDAR1 phospho-Ser890 (Cell Signaling #3381); NMDAR2A (1∶1000, ab14596); NMDAR2A phospho-Y1325 (1∶300, ab16646); CaMKII (1∶3000; BD Transduction labs 611292); CaMKII phospho-Thr286 (1∶2000; Cell Signaling 3361); PKCγ (1∶1000; Abcam ab4145). Akt (1∶1000; Cell Signaling 4691); Akt phospho-Thr308 (1∶1000; Cell Signaling 2965); Akt phosphor-Ser473 (1∶1000; Cell Signaling 4060); nNOS (1∶1000; Santa Cruz SC-1025); nNOS phospho-Ser1417 (2 µg/mL; Abcam ab5583); nNOS phospho-Ser847 (1 µg/mL; Abcam ab16650) and Actin (1∶3000; Stressgen, CSA-400). Anti-histidine triad nucleotide binding protein 1 (HINT1) antibody was raised in rabbits (Immunostep, Spain) against the peptide sequence GYRMVVNEGADGGG (aa 93–106).

The 1C8 mouse monoclonal antibody (IgM) was used to detect phosphoserines (Calbiochem #525281). The antibodies were diluted in TBS + 0.05% Tween 20 (TTBS) and incubated with the PVDF membranes for 24 h at 6°C. Primary antibodies were detected using corresponding secondary antibodies conjugated to horseradish peroxidase (diluted 1∶10,000 in TTBS). Antibody binding was visualized with an Immobilon Western Chemiluminescent HRP substrate (Millipore #WBKLS0100). Chemiluminescence was recorded with a ChemiImager IS-5500 (Alpha Innotech, San Leandro, California). Assays were performed in replicates of two or three on samples obtained from independent groups of mice. The results were consistent across all replicates.

## Results

### Regulation of Akt-nNOS pathway by morphine

**Figure 1:**
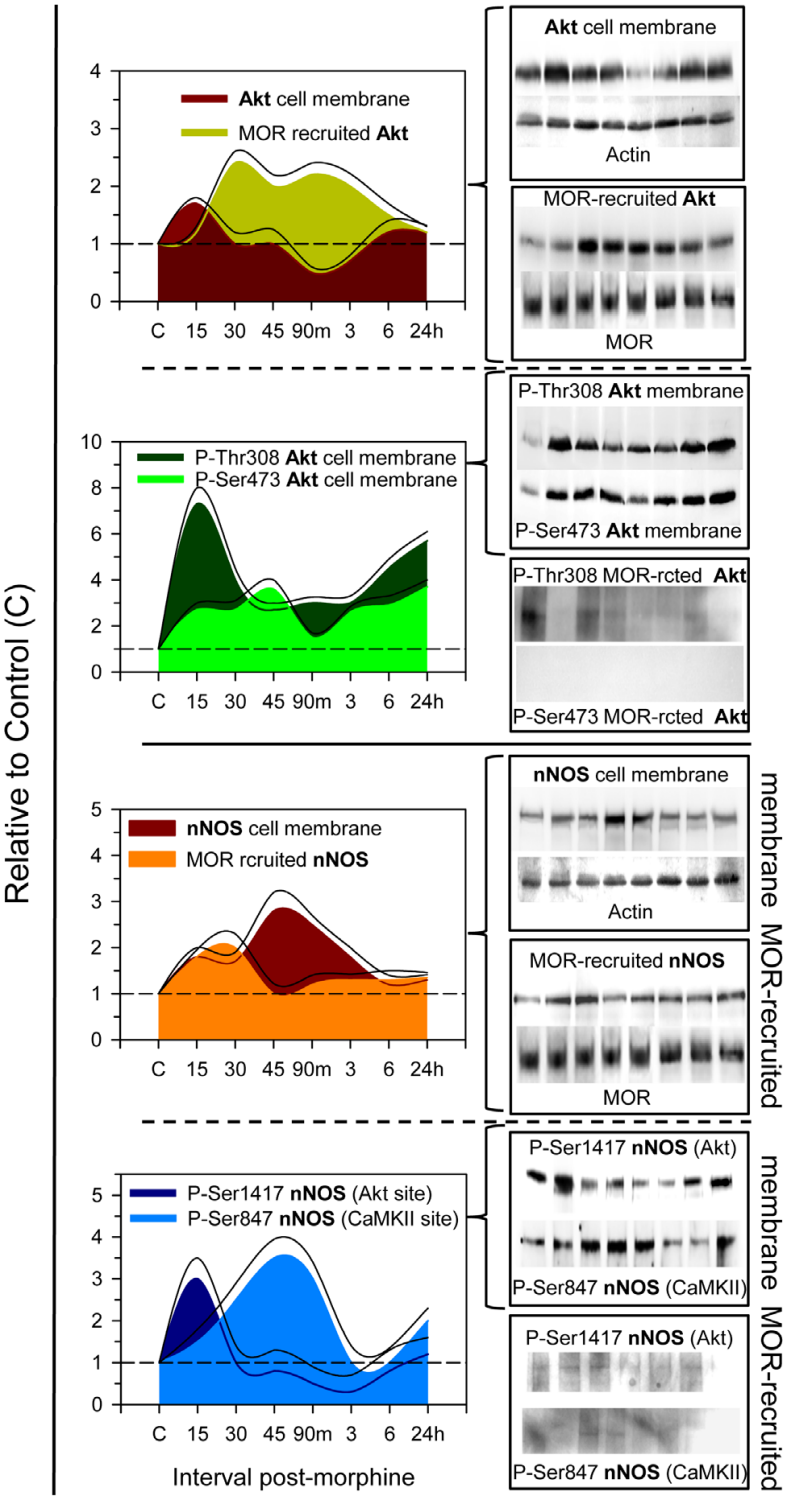
Control of Akt-nNOS pathway by the action of morphine on MORs. 10 nmol morphine was icv-injected into CD1 mice. At various intervals post-injection, the mice were sacrificed and the PAG structures were collected. The presence of Akt and nNOS in the PAG synaptosomal membrane was determined. Also, the association of these proteins with the MOR was evaluated. At each time point studied, the PAG structures from four to six mice were pooled. MOR proteins were immunoprecipitated and the co-precipitated Akt and nNOS were evaluated. Immunosignals (average optical density of the pixels within the object area/mm^2^; Quantity One Software, BioRad) were expressed as the change relative to the control animals (attributed an arbitrary value of 1). Each data point represents the mean of three assays performed on PAG samples obtained from independent groups of mice. Data are presented as the area below the curve (Sigmaplot v11), the upper curve indicates the +SEM. For comparison, we have superimposed the control levels of the pair of curves being compared. *Upper panel:* The effect of morphine on the presence of Akt in the synaptosomal PAG membrane and the MOR environment. The presence of activating Thr308 and Ser473 phosphorylations in the membrane-associated Akt. Insets: Representative western blots of these data. An absence of phosphorylation in Akt recruited to the MOR was shown but not plotted. MOR-rcted stands for MOR-recruited. *Lower panel*: A similar study of nNOS was carried out. Enzyme levels associated with PAG synaptosomal membranes and MORs were determined. The presence of Akt-activating Ser1417 phosphorylation and CaMKII-inactivating Ser847 phosphorylation were also determined in membrane-associated nNOS. Insets: Representative nNOS western blots are shown. nNOS that was recruited to the MOR environment lacked Ser1417 and Ser847 phosphorylation.

To analyze the effect of morphine on Akt activity, an acute 10 nmol dose was injected icv into mice. This dose has been shown to induce MOR-mediated analgesic desensitization [51]. Animals were sacrificed at various intervals post-injection, and the synaptosomal fraction of PAG structures was obtained for immunodetection and co-precipitation studies. During the first 15 min following morphine administration, the amount of membrane-associated Akt increased approximately 1.7-fold. This increase was followed by a decrease at 30 min, and after 90 min, the levels had dropped to below baseline. Hours later, Akt expression returned to control levels. The Akt pool was activated by the phosphorylation of Thr308 and Ser473 residues (Fig. 1, upper panel). Interestingly, 30 min to 3 h post-morphine, Akt was also recruited to the MOR. The pattern of Akt and MOR co-localization was the inverse to the amount of Akt found in the membrane. Importantly, Akt proteins recruited to MOR lacked phosphorylation on both the Thr308 and Ser473 residues.

There was a transient increase in nNOS in the synaptosomal membrane 45 min to 3 h post-morphine. Afterwards, nNOS returned to levels observed prior to the opioid challenge. These changes corresponded with a decrease in the association of nNOS with the MOR during this particular interval (Fig. 1, lower panel). Similar to Akt, membrane-associated nNOS had regulatory phosphorylation at Ser1417 and Ser847, but phosphorylation was almost undetectable for MOR-associated nNOS. Membrane-associated nNOS was phosphorylated by Akt on Ser1417. This phosphorylation activates and enhances the production of nitric oxide (NO) in the presence of Ca^2+^-CaM [38]. This activation, however, is transitory and is followed by an inactivating phosphorylation at Ser847, a CaMKII site [52]. Naloxone, an opioid antagonist, inhibits the effects of morphine on Akt-nNOS activation, indicating the involvement of MORs (not shown).

### The HINT1 protein drives the recruitment of Akt and nNOS to the MOR

**Figure 2:**
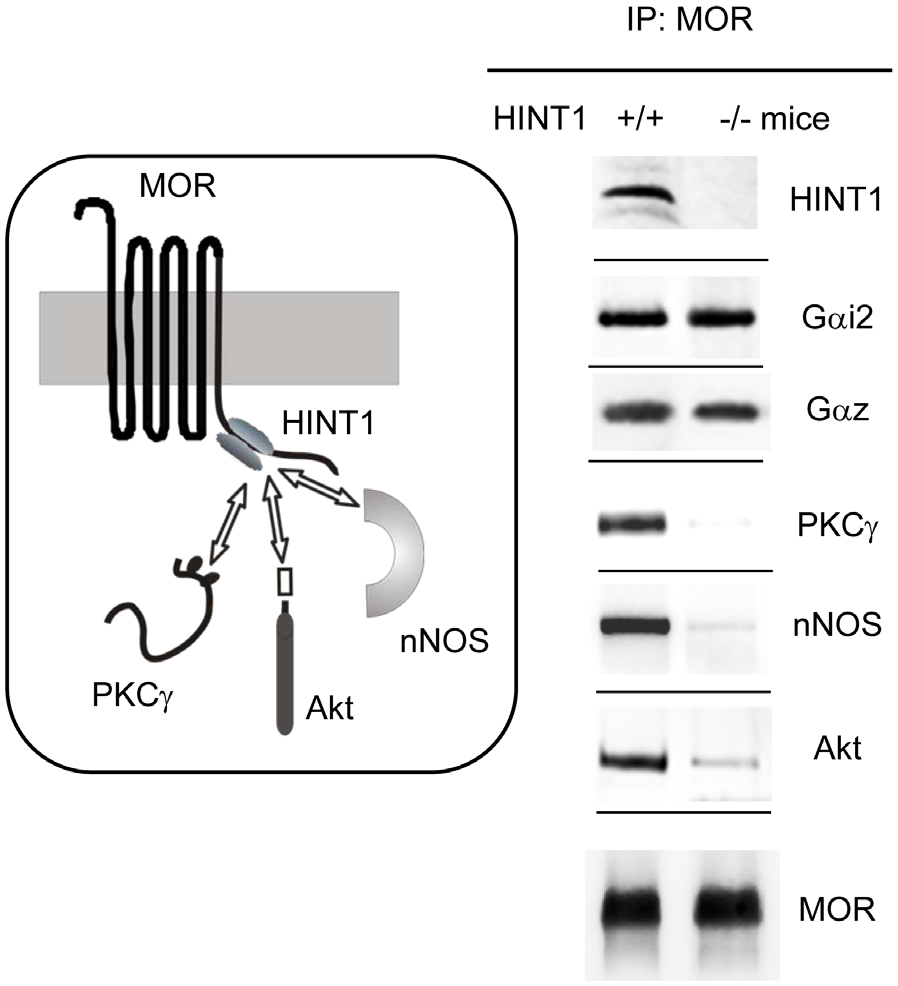
HINT1 protein binds to a series of signaling proteins at the C terminus. PAG synaptosomal membranes prepared from HINT1 (−/−) 129 SvJ mice were used to immunoprecipitate MORs and the association of the signaling proteins with MORs was studied. Signals corresponding to the MOR in HINT1 (−/−) and (+/+) mice were paired and co-precipitated proteins were directly compared. These assays were repeated twice on samples obtained from different mice. The results were consistent across replicates.

In neural cells, the C terminus of the MOR interacts with a signaling complex formed by HINT1 and RGS proteins members of the RGS-Rz subfamily. The HINT1 protein binds directly to sequences in the MOR cytosolic C tail and also in the N terminus of the RGSZ1 and RGSZ2 proteins [27], [53]. Morphine-induced MOR activation recruits PKC, primarily PKCγ, to this complex, where it is activated by diacylglycerol (DAG). DAG is generated by the action of Gβγ dimers on PLCβ [11]. In the present study, we have evaluated whether the observed association between MORs, Akt and nNOS depends on the presence of the HINT1-RGSZ complex. MORs immunoprecipitated from PAG synaptosomes isolated from HINT1 (−/−) 129SvJ mice were associated with regulated G proteins, but not PKCγ, Akt or nNOS (Fig. 2). PKCγ binds directly to the HINT1-RGSZ complex through NMDAR-nNOS generated zinc ions [11].

### MOR enhances NMDAR function via Akt-nNOS

**Figure 3:**
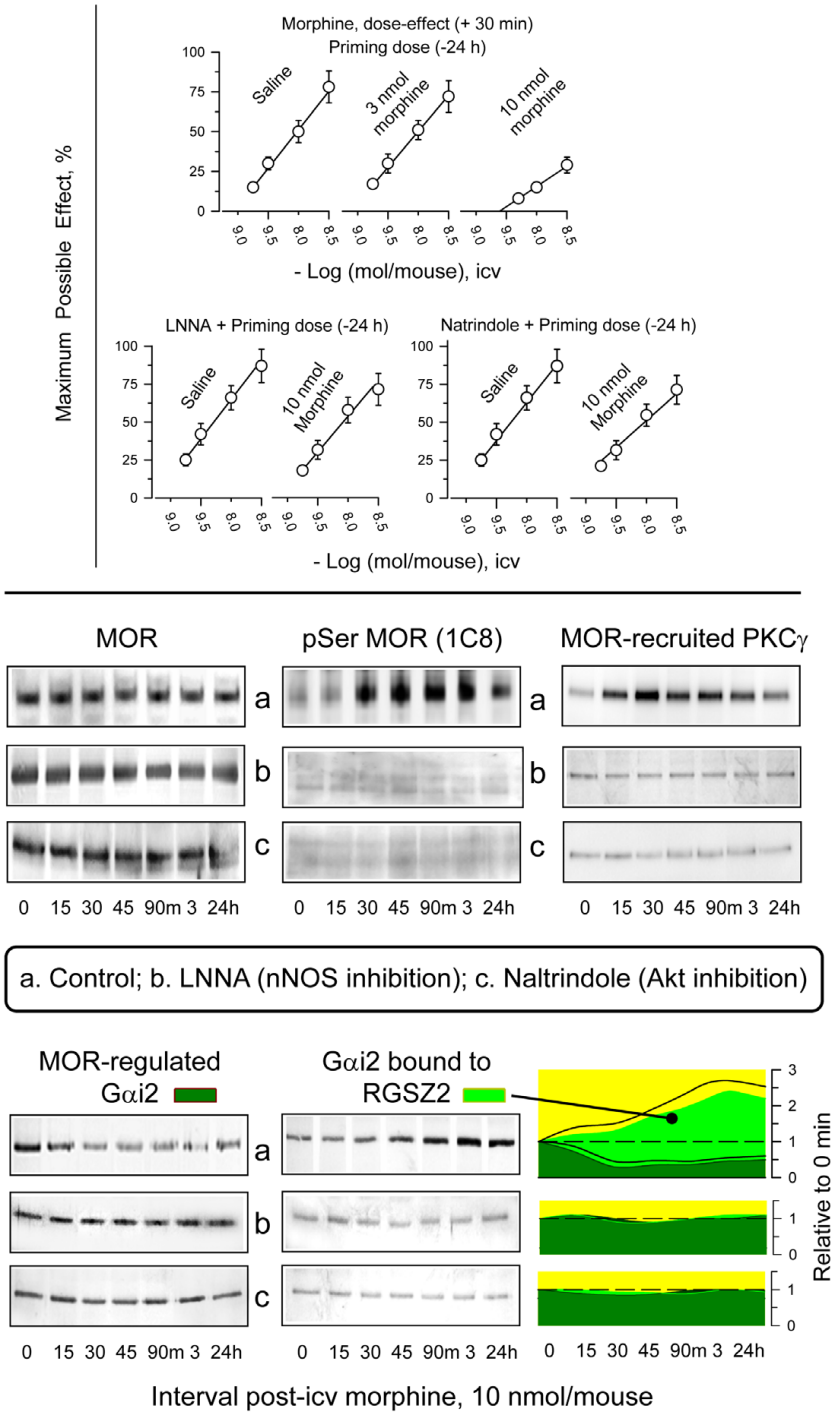
The Influence of Akt and nNOS in the production of supraspinal analgesia and changes in MOR-associated transduction as a result of morphine exposure. *Upper panel*: CD1 mice were icv-injected with increasing doses of morphine and antinociception was monitored with the warm water (52°C) tail-flick test. Data correspond to the peak-effect interval at 30 min post-injection and are presented as dose-response curves. Individual values represent the mean ± SEM from a group of eight mice. The development of morphine-induced single-dose tolerance and its pharmacological rescue were studied 24 h after receiving a priming dose of 3 nmol or 10 nmol morphine. The analgesic effect of increasing test doses of morphine was then evaluated at 30 min post-injection. The nNOS inhibitor, LNNA (7 nmol), and the mixed Delta-opioid receptor antagonist/Akt inhibitor, naltrindole (1 nmol), were icv-injected prior to the 10 nmol morphine priming dose. LNNA was injected 30 min before the priming dose, whereas naltrindole was administered 90 min before the priming dose. *Lower panel*: LNNA and naltrindole were administered to the CD1 mice prior to the 10 nmol morphine priming dose. Mice were sacrificed at the indicated time points. For each time point studied, PAG structures from four to six mice were pooled. The MOR was immunoprecipitated and serine phosphorylation was evaluated (see Materials and Methods). Co-precipitation of Gαi2 subunits with MORs and RGSZ2 proteins was also evaluated. Further data and details are shown in Fig. 1.

The icv administration of morphine produces a dose-dependent antinociceptive effect in the tail-flick test that peaks 30 min after the injection. In CD1 mice, the administration of a 10 nmol opioid dose elicits a profound decrease in response to successive doses of morphine [46]. This tolerance was present when the dose-response curve of morphine was assessed 24 h after the animals had received the 10 nmol morphine priming dose. The ED50 (nmol icv morphine/mouse) was 4.84 for control mice (95% confidence limits 3.63–6.43). Prior studies have shown that tolerance to morphine can be attenuated by nNOS inhibitors and the delta-opioid receptor antagonist, naltrindole [37]. Naltrindole, however, has recently been described as a cell permeable inhibitor of the Akt signaling pathway [54]. When mice were pre-treated with 10 nmol of morphine, they showed a profound tolerance with an ED50 >10 nmol/mouse. If mice were injected 30 min before the priming dose with 7 nmol of the competitive nNOS inhibitor LNNA, or 90 min before the priming dose with 1 nmol naltrindole, a delta-opioid receptor antagonist [55] and Akt inhibitor [54], morphine tolerance did not develop (Fig. 3, upper panel).

A single dose of 10 nmol morphine does not promote internalization of neural MORs [46]. Instead, the desensitizing effects of morphine are mediated by the phosphorylation of serine residues on the cytosolic receptor sequences as well as by the reduction of some of the regulated G proteins. During its analgesic time-course, a 10 nmol morphine dose brings about the progressive transfer of MOR-regulated Gα subunits towards the control of RGS proteins (e.g., the RGSZ2) [46], [50]. This impairment of MORs to regulate their transduction results in morphine tolerance. In agreement with previous reports, ex vivo assays showed the following after a morphine challenge: the presence of MORs in the membrane surface remained constant, morphine increased the recruitment of PKCγ at the MOR, which became phosphorylated, and a gradual loss of regulated G proteins was increasingly associated with RGSZ2 proteins [11], [46]. Administration of LNNA or naltrindole before morphine abolished the development of analgesic tolerance, prevented the recruitment of PKCγ at the MOR, inhibited phosphorylation of the opioid receptor and interfered with the transfer of G proteins from MOR control toward RGSZ2 control (Fig. 3, lower panel).

### Regulatory loop MOR-Akt-nNOS-NMDAR-CaMKII-MOR

**Figure 4:**
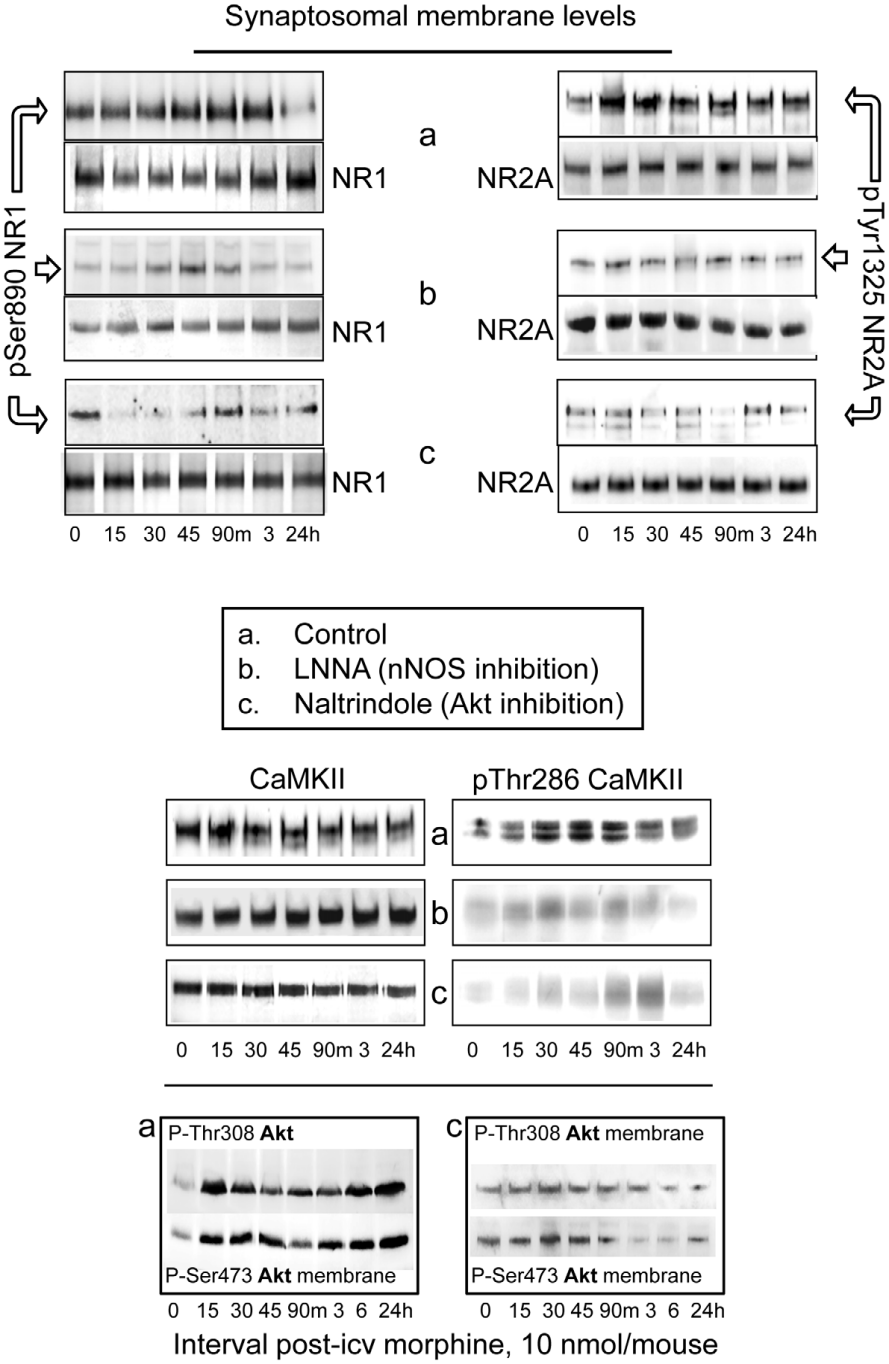
The Influence of the Akt-nNOS pathway on morphine-induced NMDAR potentiation. *Upper panel*: The influence of 10 nmol icv morphine on NMDAR activity was evaluated. The levels of NMDAR1 and of NMDAR2A subunits and activating phosphorylations at NR1 Ser890 (PKC site) and NR2A Tyr1325 (Src site) were monitored in the CD1 PAG synaptosomal membranes during the post-morphine intervals indicated. The presence of the Thr286 autophosphorylation of NMDAR-activated CaMKII was also determined. Administration of LNNA or naltrindole before morphine greatly reduced NR1, NR2A and CaMKII phosphorylation. *Lower panel*: Naltrindole decreased Akt/PKB activating phosphorylation. Please see Materials and Methods for further details.

One of the main targets of GPCR-regulated Src is the cytoplasmic tail of the NR2A and NR2B subunits [4], [56]. The 10 nmol morphine dose promoted PKC-mediated phosphorylation of NMDAR1 Ser890 and Src-mediated phosphorylation of NMDAR2A Tyr1325. Both phosphorylations were previously found to enhance NMDAR function [29], [37]. Indeed, these changes are associated with the activating Thr286 autophosphorylation of CaMKII. Again, administration of LNNA or naltrindole before morphine prevented the phosphorylation of NMDAR subunits and the activation of CaMKII as well (Fig. 4). Notably, naltrindole reduced phosphorylation of Akt on Thr308 and Ser473. Therefore, independent of its antagonist activity at the delta-opioid receptor, naltrindole may act as a cell permeable inhibitor of Akt [54].

**Figure 5:**
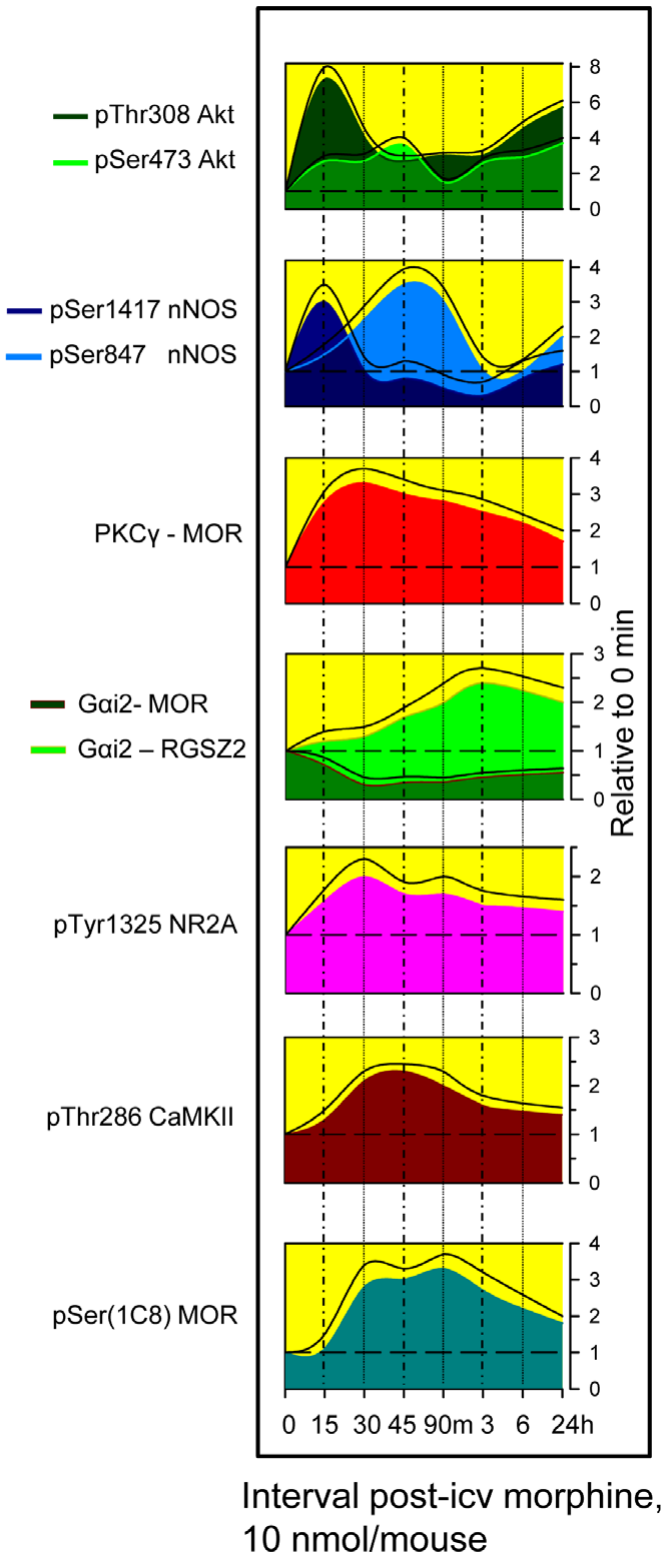
Chronology of morphine-triggered changes in MOR-regulated signaling proteins. 10 nmol morphine was icv-injected into the CD1 mice at time 0. The data, the mean of three assays performed on PAG samples obtained from independent groups of mice, are presented as the area below the curve (Sigmaplot v11) and the upper curve indicates +SEM. In instances where a pair of curves is plotted together, the control levels have been superimposed. Details are provided in the legends for Figs 1, 3 & 4.

In neuronal cells, the glutamate NMDAR is important for both the control and desensitization of the MOR [12], [13], [30]. Morphine-activated MOR enhances NMDAR-mediated glutamate responses [14], bringing about the activation of CaMKII [15]. CaMKII, in turn, inhibits MOR signaling [30]. Fig. 5 shows the chronology of a series of morphine-triggered events that participate in the regulation of MOR signaling and development of analgesic tolerance. Considering that time 0 is the moment of morphine icv-injection, the earliest event observed was Akt activation, which was immediately followed by the activating phosphorylation of nNOS. Shortly afterwards, PKCγ was recruited to the MOR, NR2A was phosphorylated and the presence of Gαi2 decreased at the MOR. These events coincided with a decrease in the activation of Akt via pThr308, the appearance of active pThr286 CaMKII and subsequent reduction of nNOS activity via an increase of pSer847 and dephosphorylation of pSer1417. CaMKII activation was also followed by the phosphorylation of MOR and the transfer of Gα subunits from the MOR toward the RGSZ2 protein. For longer intervals (6 h and 24 h post-morphine), most of these changes were still present, although the magnitude was reduced: Akt recovered some activating phosphorylation, PKCγ was still observed at MORs and the levels of pTyr1325 NMDAR2 and of pThr286 CaMKII decreased almost to the control level. The phosphorylation of the MOR, however, was still present, as was the association between Gα subunits and RGSZ2 proteins.

## Discussion

Our results indicate that in PAG morphine binding to MORs elicits sustained potentiation of NMDAR function through the activation of the Akt/PKB-nNOS pathway upstream of PKCγ and Src. Akt activation was observed a few minutes after morphine administration and was rapidly transmitted to the nNOS. This indicates that the MOR is coupled to the production of NO via the Akt-nNOS pathway. Notably, early Akt-nNOS activation was immediately followed by a decrease in its strength prior to the 30 min interval, the time when the peak analgesic effect of morphine was reached. A series of regulatory mechanisms, such as the recruitment of Akt to the MOR, were triggered to control neuronal NO levels. Moreover, transient Akt-activating phosphorylation of nNOS Ser1417 [38] was followed by a robust increase in CaMKII-mediated inactivating phosphorylation of nNOS Ser847 [57]. As observed for Akt, the morphine-activated MOR also recruited inactive nNOS to reduce the stamina of the Akt-nNOS pathway. Therefore, morphine triggers a series of mechanisms that efficiently control the production of NO, which could help prevent cellular damage caused by NO overproduction. In fact, morphine exerts a protective effect in peroxynitrite-induced apoptosis of neural cells, a mechanism involving G proteins and PI3K [58]. Morphine also protects primary rat astrocytes from glutamate-induced death [59].

In a previous study, we reported that morphine promotes the recruitment of PKCγ to the HINT1-RGSZ complex at the MOR carboxy-terminus [11]. The MOR–PKCγ association involves the cysteine rich domains in the regulatory C1 region of PKCγ, NO, free zinc ions, HINT1 and RGSZ proteins. The binding of inactive Akt and nNOS to the MOR also involves the HINT1 protein. However, it appears to be independent of zinc ions and may require a third partner protein. The precise protein interactions between HINT1 and Akt or nNOS are presently being explored.

A number of GPCRs regulate NMDAR activity through the action of kinases such as Src and PKC [3], [37]. PKC is involved in MOR-generated signals that promote sustained potentiation of NMDAR-mediated glutamate responses [15]. Through its recruitment to the MOR, PKCγ is allocated close to potential substrates where DAG promotes its activation. The presence of zinc ions and DAG in the regulatory domain of PKC extends the activation period of this kinase [11], [48]. This characteristic contributes to the long-term NMDAR-mediated desensitization of MORs that is observed even after the antinociceptive effects of a desensitizing dose of morphine have disappeared. Thus, the MOR increases calcium ion permeation through the NMDAR via PKCγ, Src tyrosine kinase and Gi proteins [29]. Afterwards, CaMKII is activated and inhibits MOR signaling [30]. Inhibition of nNOS by LNNA prevents morphine tolerance [37], [60], sustained potentiation of NMDAR function and subsequent CaMKII activation. Notably, naltrindole reduced Akt activation, suggesting that it can act as a cell permeable Akt inhibitor in vivo [54]. Another possibility is that naltrindole could preserve morphine analgesia by acting as an antagonist to delta-opioid receptors [37]. Naltrindole, however, was useful in demonstrating the role of Akt as an initiator of morphine tolerance via NMDAR potentiation.

**Figure 6:**
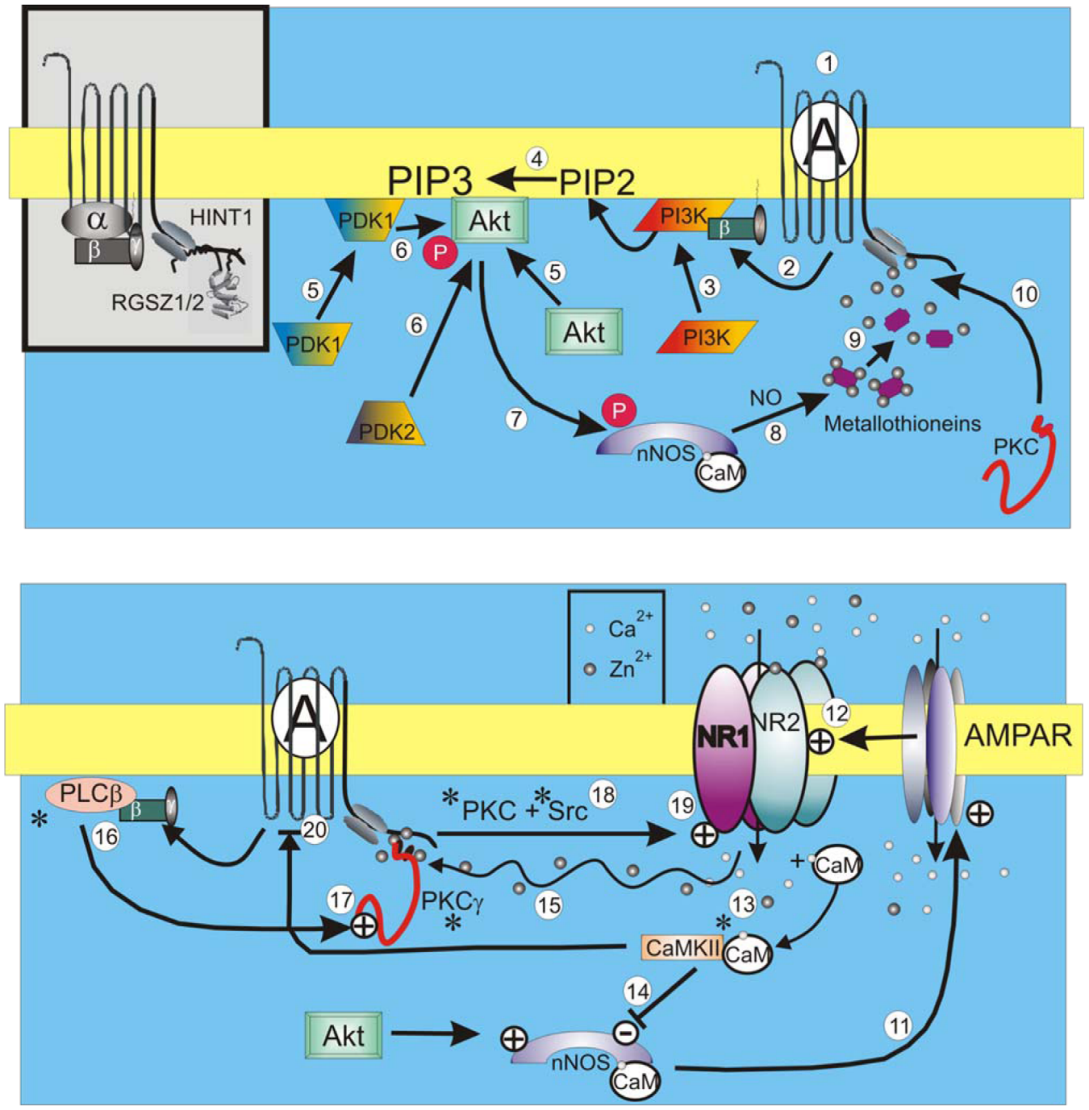
A diagram summarizing the proposed connection between MOR and NMDAR and the role of the Akt-nNOS pathway in cross-regulation. See Discussion for details.

The sequence of these morphine-triggered events is described in Fig. 6. The productive binding of morphine to MORs induces the release of free Gβγ dimers from activated Gαo/iGTP subunits (1). Agonist-freed Gβγ dimers remain attached to the membrane through lipids that are covalently-bound to the N-terminus of the Gγ subunit (2). Binding to heterodimeric PI3K translocates this lipid kinase to the membrane (3) in contact with its substrates [61]. Membrane-associated PIP2 is converted into PIP3 by PI3K (4) and recruits the PH domain of Akt to the membrane (5) where it is phosphorylated by Phosphoinositide Dependent protein Kinase 1 (PDK1), which also has a PH domain at its C-terminus. PDK1 is recruited to the membrane by PI3K-generated PIP3 (5) where it phosphorylates and activates AGC kinases, such as PKC and Akt, through phosphorylation of Akt Thr308 (6). Akt activation is dependent on phosphorylation of two sites: PDK1 phosphorylation of Thr308 in the kinase activation loop and PDK2 phosphorylation of Ser473 at the C-terminus (6). Ser473 phosphorylation is dependent on PI3K. However, the identity of the PDK2 acting on Thr308 is still controversial. Phosphorylation of both Ser473 and Thr308 residues contribute to Akt activation [32]. Akt induces a rapid and transient phosphorylation of nNOS Ser1417 (7) resulting in increased NO production (8), release of zinc ions from metallothioneins (9) and recruitment of PKCγ to the HINT1-RGSZ complex at the MOR C-terminus (10). In the postsynapse, the regulation of nNOS is dependent on the balance between activating and inactivating phosphorylation. Akt activates nNOS, whereas CaMKII inactivates it [38], [57], [62], [63].

Physiological stimulation brings about the release of glutamate and zinc from synaptic vesicles. Zinc ions enter postsynaptic neurons through specific ionotropic receptors such as NMDA receptors, AMPA receptors and voltage-gated calcium channels [64]. When nNOS-generated NO operates on AMPA receptors (11) and potentiates NMDAR function (12), the levels of permeated calcium and zinc increase. Ca^2+^-CaM then increases and activates CaMKII (13) that, in turn, acts on nNOS Ser847 to reduce Ca^2+^-CaM binding and NO production (14). The rapid and transient MOR-dependent Akt-mediated nNOS serine1417 phosphorylation followed by sustained CaMKII phosphorylation of serine847 has also been observed for NMDAR-activated PI3K-Akt [38]. As a result of this regulation, NO production is only transiently activated. During intervals of diminished NO production due to the loss of Akt activity and the action of CaMKII on nNOS, the zinc ions required for PKCγ to bind to the MOR-HINT1 must come from another source. NMDAR-permeated zinc takes control over the recruitment of PKCγ to the MOR, (15) where the action of morphine on PLCβ (16) promotes PKCγ activation (17). Antagonism of NMDARs elicits decreased PKCγ recruitment to postsynaptic sites [65]. The concerted action of PKC and Src (18) result in sustained NMDAR potentiation (19). This MOR to NMDAR activating loop is downregulated by CaMKII-mediated negative feedback to inhibit MOR (20).

CaMKII, a Ca^2+^-CaM-activated protein kinase that is highly abundant in the CNS, has emerged as a critical factor in the NMDAR to MOR section of the regulatory loop. There is convincing evidence that supports the role of CaMKII in the development of morphine tolerance: CaMKII activity is increased in brain areas of animals with morphine tolerance [66], [67] and administration of KN62 and KN93, specific CaMKII inhibitors, significantly reduces morphine tolerance in mice and rats [68]–[70]. Furthermore, antisense oligonucleotides that specifically decrease the expression of CaMKII attenuate morphine tolerance and dependence [69]. Activated CaMKII produces a prolonged reduction of nNOS activity [52], and reduces MOR signaling [30]. PKC activity also participates in AMPA-mediated potentiation of NMDAR function [71], [72] and is critical for the development of morphine tolerance. Soon after PKCγ translocates to the MOR, activated Gα subunits are progressively transferred under the control of certain RGS proteins by a PKC-dependent mechanism [46], [48]. These processes occur before repeated morphine administration leads to internalization and recycling of the neural MOR, a process mediated by GRKs and β-arrestins [46].

A growing body of evidence indicates that MORs and NMDARs coexist in the postsynapse, explaining the relationship between opioids and glutamatergic NMDARs. These receptors are co-expressed in areas of the CNS, such as in PAG neurons [37]. Both MOR and NMDAR are modulated by spinophilin (neurabin II) [73], [74], a dendrite spine marker [75]. Moreover, at the ultrastructural level, both receptors show co-localization [76], suggesting that MOR and NMDAR could physically interact in the manner described for GPCRs such as D1, mGlu5a and the NR1 subunit of the NMDAR [77]–[79]. In fact, results generated by several studies indicate that most experimental approaches that interfere with the development of morphine analgesic tolerance can be assembled into a common regulatory machinery, which connects MOR and NMDAR function [37]. Our analyses of the on-off regulating phosphorylation of signaling proteins indicate that they work in a concatenated manner, with every step depending on the completion of the preceding step. This regulation integrates the positive effects of PI3K, Akt, nNOS, PKC, Src, NMDAR and CaMKII on MOR inhibition. The efficacy of the described treatments in preventing and rescuing morphine analgesia from tolerance can be explained by linking early events with those responsible for sustained NMDAR potentiation and subsequent CaMKII action on MORs. Upon inhibition of these early processes (e.g., Akt or nNOS), the subsequent steps responsible for NMDAR sustained potentiation and MOR tolerance do not occur. If PKC is inhibited or the NMDAR is blocked, then the early steps fail to transmit signals for activation and no tolerance develops. Similarly, the inhibition of CaMKII prevents MOR phosphorylation and reduces AMPAR function [80]. As a result, a less pronounced morphine tolerance develops [30]. In summary, early activation of Akt and nNOS initiates the PKCγ-Src pathway responsible for sustained potentiation of NMDAR-CaMKII function. This sustained potentiation results in MOR inhibition and produces an analgesic tolerance to morphine.
